# Heparin versus 0.9% sodium chloride intermittent flushing for preventing occlusion in newborns with peripherally inserted central catheters: A systematic review protocol

**DOI:** 10.1371/journal.pone.0278068

**Published:** 2022-12-30

**Authors:** Alice Passos do Nascimento, Kleyton Santos de Medeiros, Ana Paula Ferreira Costa, Ayane Cristine Sarmento, Giovanna Karinny Pereira Cruz, Ana Katherine Gonçalves, Nilba Lima de Souza, Maria de Lourdes Costa da Silva

**Affiliations:** 1 Januário Cicco Maternity School, Federal University of Rio Grande do Norte (UFRN), Natal, Brazil; 2 Institute of Teaching, Research and Innovation, League Against Cancer, Natal, RN, Brazil; 3 Health Sciences Postgraduate Program, Federal University of Rio Grande do Norte (RN), Natal, RN, Brazil; 4 School of Health, Federal University of Rio Grande do Norte, Natal, Brazil; 5 Department of Obstetrics and Gynaecology, Federal University of Rio Grande do Norte (UFRN), Natal, RN, Brazil; 6 Department of Nursing, Federal University of Rio Grande do Norte (UFRN), Natal, Brazil; 7 Postgraduate Program in Applied Sciences to Women’s Health, Federal University of Rio Grande do Norte (RN), Natal, RN, Brazil; Beijing University of Chinese Medicine, CHINA

## Abstract

**Background:**

Mechanical factors are primary complications that justify early removal of a peripherally inserted central catheter, and thrombotic catheter occlusion is the most critical mechanical complication associated with loss of device functionality. Studies have investigated these factors in adult patients, but findings are not directly applicable to newborns. Therefore, systematic reviews focusing on this population are necessary for consolidated evidence to aid clinical practice.

**Aims:**

This study aims to evaluate the efficacy of intermittent heparin washing versus 0.9% sodium chloride solution for preventing occlusion in newborns with peripherally inserted central catheters.

**Methods:**

We will use the PubMed, Embase, Scopus, Web of Science, Cochrane Central Register of Controlled Trials, CINAHL, and Clinical Trial Databases for article search, without language or publication periods restrictions. Randomized clinical trials evaluating the use of intermittent heparin washing versus 0.9% sodium chloride solution in newborns with peripherally inserted central venous catheters will be included. The primary outcome will be peripherally inserted central catheter occlusion. Two reviewers will independently screen the studies, based on the inclusion criteria, extract the data for each included study and assess the risk of bias using the Cochrane risk of bias tool. The data will be synthesized using the Review Manager software (RevMan 5.4.1). To classify the strength of the evidence of results, we will use the Grading of Recommendations Assessment, Development and Evaluation Working Group (GRADE). The review was registered with PROSPERO (registration number CRD42021281509).

**Expected results:**

We expect that this study would reveal the best method for preventing catheter occlusion in newborns with peripherally inserted central catheters.

## Introduction

Inserting a peripherally inserted central catheter (PICC) is common in clinical practice. This device has become indispensable in neonatal intensive care units (NICU) because it can be conveniently inserted at the bedside without surgical intervention [1–6]. The indication is primarily for premature babies, babies with deficient birth weight, or critically ill infants when long-term intermediate venous access is required for medications and fluid therapy, parenteral nutrition, or blood collection, depending on the catheter caliber. However, peripheral venous devices have short permeability, and the insertion sites may be exhausted during prolonged therapy [1, 2, 4].

Therefore, early insertion of the PICC is essential to reduce postnatal problems, such as portal hypertension induced by poor positioning of the umbilical catheter. Besides avoiding problems such as infections and pain caused by repeated punctures, it also increases the survival rates of babies with low birth weight [2, 3].

The advantages of this type of venous access include elimination of intrathoracic disorders (pneumothorax and hemothorax) caused by the insertion of central venous catheters, especially in the subclavian vein. Additionally, the latter could reduce costs because it can be performed outside the operating room without general anesthesia [3, 7].

PICC-related complications include infections, phlebitis, venous thrombosis, and mechanical factors such as catheter displacement, leakage, rupture, and occlusion. The latter can be classified as thrombotic or non-thrombotic and is mainly related to the precipitation of medications inside the device. Moreover, there is a significantly higher risk of catheter occlusion in patients with PICCs with a lumen smaller than 2 French, which is the most commonly used in neonatology. Such complications can result in increased health-care costs owing to increased length of stay in the NICU and newborn injuries [2, 4–6, 8–12].

Several international guidelines provide recommendations for the care of central venous devices, especially for occlusion prevention. For this, intermittent flushing with saline solution is recommended before and after the infusion of medicines. However, as few studies specifically address PICCs, especially in pediatric and neonatal populations, these recommendations are based mainly on adult studies and studies dealing with different types of central venous catheters [8–12].

When the catheter is not in continuous use, occlusion may be reduced by intermittent catheter washing with saline or heparin solution (50–100 U/ml) corresponding to the catheter volume. The solution is removed before reusing the catheter for infusion of other solutions [7].

However, heparin and other thrombolytic agents should be used with extreme caution. Activated partial thromboplastin time should be closely monitored, and protamine sulfate should be available in case there is an event of bleeding. Clinical experience with other thrombolytic agents in newborns is limited and has demonstrated inconsistent efficacy [1, 7].

Therefore, this study aims to evaluate the effectiveness of flushing with heparin when compared with that of flushing with saline solution in maintaining PICCs.

## Materials and methods

The systematic review protocol was designed based on the Preferred Reporting Items for Systematic Reviews and Meta-Analysis Protocols (PRISMA-P) [13, 14]. The review was registered with PROSPERO (registration number CRD42021281509).

### Ethical considerations

Secondary data were used in this study, so obtaining approval from the ethics committee was not necessary.

### Inclusion criteria

Randomized clinical trials and quasi-randomized clinical trials evaluating the use of intermittent heparin flush (any dose) compared with that of 0.9% sodium chloride solution (saline) in newborns with a PICC will be included in the sample. Will be considered newborns the children < 28 days according to World Health Organization (WHO). Newborns of any gestational age will be included in this study.

### Exclusion criteria

Published but not peer-reviewed articles will be excluded from the review. Observational studies, review articles, reports, and case series will also be excluded. Studies evaluating the use of other drugs associated with heparin as an intervention in children (>28 days of age) will also be excluded.

### Patient, intervention, comparison, outcome, and time (PICOT) strategy

The PICOT strategy will be used as follows: population—newborn; intervention/exposure—intermittent flush with heparin; comparator/control—0.9% sodium chloride solution (saline); outcomes—PICC occlusion; and types of study to be included—randomized clinical trials and quasi-randomized clinical trials.

### Primary outcome

PICC occlusion which is determined by the inability to infuse fluids through the catheter.

### Secondary outcomes

The secondary outcomes will be duration in the PICC days; incidence of catheter removal/reinsertion, and PICC-related thrombosis.

### Search strategy

A comprehensive search of the following databases was conducted: PubMed, Embase, Scopus, Web of Science, Cochrane Central Register of Controlled Trials, CINAHL, and Clinical Trial Databases (www.trialscentral.org; www.controlled-trials.com, ClinicalTrials.gov). No language, publishing period restrictions or other filters will be enforced.

The terms of the Medical Subject Headings and keywords will be (Neonate OR Newborn Infants OR Preterm Infant OR Extremely Premature Infant) AND (Catheterization, central venous OR Central Venous Catheters OR Peripherally Inserted Central Catheter Line Insertion OR PICC OR vascular access device) AND (heparin OR sodium heparin) AND (sodium chloride OR saline solution, hypertonic) AND (randomized controlled trial OR controlled clinical trial). The search strategy used in PubMed is shown in Table 1.

**Table 1:** Search strategy for PubMed.

	MeSh Terms and Keywords
1	Neonate
2	Newborn Infants
3	Preterm Infant
4	Extremely Premature Infant
5	OR / 1–4
6	Catheterization, central venous
7	Central Venous Catheters
8	Peripherally Inserted Central Catheter Line Insertion
9	PICC
10	Vascular access device
11	OR / 6–10
12	heparin
13	sodium heparin
14	OR / 12–13
15	sodium chloride
16	saline solution, hypertonic
17	OR / 15–16
18	5 AND 11 AND 14 AND17

### Data collection and analysis

#### Study selection

**Fig 1:**
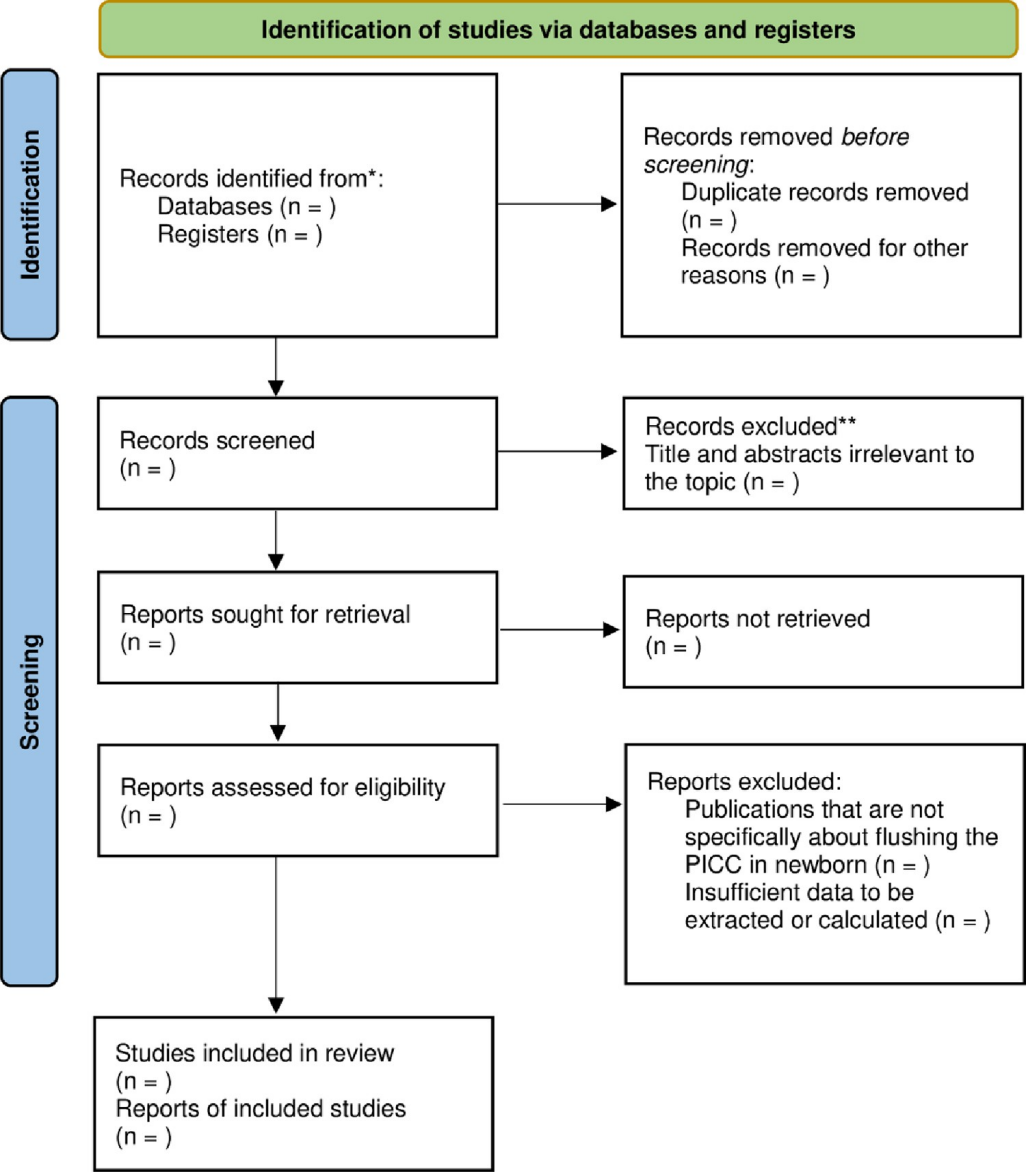
PRISMA flow diagram for systematic review and meta-analysis.

After searching the databases, all identified articles will be exported to the Rayyan software, and duplicates will be removed. Based on the inclusion criteria, titles and abstracts will be read independently by at least two reviewers (APN and KSM). The full texts of these potentially eligible studies will be independently retrieved and considered for eligibility by the two reviewers (APN and KSM). Only studies identified by both reviewers will be ultimately included in the systematic review. In case of a discrepancy, a third reviewer (MLCS) will make the final decision for inclusion. We will maintain a record of the reasons for excluding clinical trials at all stages of review. The results of the inclusion or exclusion of studies will be reported using the PRISMA flowchart (Fig 1).

#### Data extraction

A standardized data extraction form will be developed and tested (S1 Appendix). Two reviewers will independently extract the data for each included study, and any subsequent discrepancies will be resolved through discussion with a third reviewer. The extracted data will include information such as authors, year of publication, type of study, main objectives, population (inclusion criteria of the participants), description of interventions, monitoring of participants, variables associated with catheter maintenance, and outcome measures. In addition, the characteristics of the participants (mean age and sex) will be collected.

#### Missing data

In case of a lack of data, the authors will contact the authors or co-authors of the article in question by phone or e-mail. If information is not received, the data will be deleted from our analysis and covered in the discussion section.

#### Data synthesis

The data will be entered into the Review Manager software (RevMan 5.4.1), which allows the user to enter protocols, complete revisions, including text, study characteristics, comparison tables, study data, and perform meta-analyses. We will evaluate heterogeneity between studies using the I2 statistics (<25%, low heterogeneity, 25%-50%, moderate heterogeneity, and > 50%, high heterogeneity). The fixed effects models will be used, except when significant heterogeneity existed in included studies (I2>50%). The odds ratio (OR) with 95% confidence intervals (CI) will be performed to estimate the corresponding risk. Dichotomous data from each of the eligible studies were combined for meta-analysis using the Mantel/Haenszel model.

In this review, we will use only clinical trials. However, we will conduct a subgroup analysis for the different doses and methods of application of heparin and frequency of washings of heparin or saline. A two-sided p-value <0.05 will be considered statistically significant. If it is not possible to perform a meta-analysis, a qualitative analysis will be carried out.

#### Quality assessment

Two reviewers (APN and KSM) will independently assess the risk of bias in the included studies using the Cochrane risk of bias tool version RoB 2 for the trials. Thus, the criteria for their judgments will be Randomization process; Deviations from intended interventions; Missing outcome data; Measurement of the outcome; Selection of the reported results; and Others bias / Overall. We will use the Shiny app-robvis as a visualization tool for generating the risk of bias figures [15, 16]. Disagreements among the two reviewers on the risk of bias in specific studies will be resolved by discussion with the involvement of a third author (MLCS). Publication bias will be assessed by inspection of the funnel plot and formal testing for asymmetry of the funnel plot using Egger’s test.

#### Assessing certainty in the findings

To classify the strength of evidence from the included data, we will use the Grading of Recommendations Assessment, Development and Evaluation Working Group (GRADE) approach. The evaluation summary will be incorporated into broader measurements to ensure judgment of the risk of bias, consistency, objectivity, and accuracy. The quality of evidence will be assessed based on the risk of bias, indirect bias, inconsistency, inaccuracy, and publication bias [17].

## Discussion

The use of PICC has become essential in neonatal clinical practice, and knowledge of the appropriate methods for its maintenance is of fundamental importance for the success of therapy [7, 12]. Knowledge of the efficacy of intermittent heparin flush in relation to that of saline solution to prevent PICC occlusion is important for clinical practice in the NICU, as it may allow the adoption of strategies that reduce the occurrence of this complication, which can lead to longer hospital stays for NB in the NICU and higher costs of treatment. Occlusion has been associated with unscheduled catheter removal [7, 12]. A study of 14,278 PICCs has suggested that occlusion affects up to 12% of devices and has therefore been associated with a high cost [3].

To aid health teams direct their preventive measures, it is important to understand which of these agents is more effective in preventing obstruction, in order to reduce the prevalence of complications, need for new punctures, and associated risk of infection. In the adult population, several studies have compared the use of heparin versus that of saline solution in the maintenance of PICC. However, the findings of adult studies is not directly applicable to pediatrics, especially for neonatology, so systematic reviews focusing on newborns are necessary to consolidate evidence to aid clinical practice [7, 12, 18].

The potential limitations of this systematic review will focus on aspects of the study design, research, and evaluation of the quality of the included studies. Additionally, a reduced number of samples and limited scans of studies may influence the reliability of the findings. Limitations related to randomized controlled trials include limited generalization of the study population and the answer to a specific research question.

## Supporting information

S1 Checklist PRISMA-P 2015 checklist. (DOCX)

S1 Appendix Data extraction form. (DOCX)

S1 File Supplementary material (GRADE). (DOCX)
