# Supplementary material for: Heparin versus 0.9% sodium chloride intermittent flushing for preventing occlusion in newborns with peripherally inserted central catheters: A systematic review protocol
Source: PLoS One. 2022 Dec 30;17(12):e0278068. doi: 10.1371/journal.pone.0278068 (PMC9803159; doi:10.1371/journal.pone.0278068)
Supplement: S1 Appendix — (DOCX) [file pone.0278068.s002.docx]

**APPENDIX S1 - DATA EXTRACTION FORM**

1. **Characteristics of the article and population**

| **Author** | **Year of publication** | **Country** | **Study design** | **Primary objective** | **Inclusion criteria** | **Sample size** | **Gestational age** | **Chronological age** |
| --- | --- | --- | --- | --- | --- | --- | --- | --- |
|  |  |  |  |  |  |  |  |  |
|  |  |  |  |  |  |  |  |  |
|  |  |  |  |  |  |  |  |  |
|  |  |  |  |  |  |  |  |  |
|  |  |  |  |  |  |  |  |  |
|  |  |  |  |  |  |  |  |  |
|  |  |  |  |  |  |  |  |  |

1. **Outcomes**

| **Intervention** | **Dose** | **Control** | **Dose** | **Occlusion incidence** | **Duration of the PICC** | **Incidence of removal** | **Incidence of reinsertion** | **PICC-related thrombosis** |
| --- | --- | --- | --- | --- | --- | --- | --- | --- |
|  |  |  |  |  |  |  |  |  |
|  |  |  |  |  |  |  |  |  |
|  |  |  |  |  |  |  |  |  |
|  |  |  |  |  |  |  |  |  |
|  |  |  |  |  |  |  |  |  |
|  |  |  |  |  |  |  |  |  |
|  |  |  |  |  |  |  |  |  |
