# Supplementary material for: Heparin versus 0.9% sodium chloride intermittent flushing for preventing occlusion in newborns with peripherally inserted central catheters: A systematic review protocol
Source: PLoS One. 2022 Dec 30;17(12):e0278068. doi: 10.1371/journal.pone.0278068 (PMC9803159; doi:10.1371/journal.pone.0278068)
Supplement: S1 File — (DOCX) [file pone.0278068.s003.docx]

| **Summary of findings:** | | | | | | |
| --- | --- | --- | --- | --- | --- | --- |
| **Heparin compared to Sodium chloride solution for Newborn with PICC** | | | | | | |
| **Patient or population:** Newborn with PICC  **Setting:** Peripherally inserted central catheter (PICC)  **Intervention:** Heparin  **Comparison:** Sodium chloride solution | | | | | | |
| Outcomes | **Anticipated absolute effects^*^** (95% CI) | | Relative effect (95% CI) | № of participants (studies) | Certainty of the evidence (GRADE) | Comments |
|  | **Risk with Sodium chloride solution** | **Risk with Heparin** |  |  |  |  |
| Occlusion | 0 per 1.000 | **0 per 1.000** (0 to 0) | not estimable | ( studies) | - |  |
| Incidence of catheter removal | 0 per 1.000 | **0 per 1.000** (0 to 0) | not estimable | ( studies) | - |  |
| Incidence of catheter reinsertion | 0 per 1.000 | **0 per 1.000** (0 to 0) | not estimable | ( studies) | - |  |
| PICC -related thrombosis | 0 per 1.000 | **0 per 1.000** (0 to 0) | not estimable | ( studies) | - |  |
| ***The risk in the intervention group** (and its 95% confidence interval) is based on the assumed risk in the comparison group and the **relative effect** of the intervention (and its 95% CI).  **CI:** confidence interval | | | | | | |
| **GRADE Working Group grades of evidence** **High certainty:** we are very confident that the true effect lies close to that of the estimate of the effect. **Moderate certainty:** we are moderately confident in the effect estimate: the true effect is likely to be close to the estimate of the effect, but there is a possibility that it is substantially different. **Low certainty:** our confidence in the effect estimate is limited: the true effect may be substantially different from the estimate of the effect. **Very low certainty:** we have very little confidence in the effect estimate: the true effect is likely to be substantially different from the estimate of effect. | | | | | | |
